# The psychological consequences of Sudden Infant Death Syndrome (SIDS) for the family system: A systematic review

**DOI:** 10.3389/fpsyg.2023.1085944

**Published:** 2023-02-23

**Authors:** Gabriella Gandino, Alessia Diecidue, Annalisa Sensi, Ester Maria Venera, Sarah Finzi, Cristina Civilotti, Fabio Veglia, Giulia Di Fini

**Affiliations:** ^1^Department of Psychology, University of Turin, Turin, Italy; ^2^SUID and SIDS Italia Onlus, Turin, Italy

**Keywords:** sudden infant death syndrome (SIDS), systematic review, family grief, psychological impact, couple grief, individual grief

## Abstract

The Sudden Infant Death Syndrome (SIDS) is a tragic and difficult experience for families. It involves not only the death of the baby but also the loss of a future as a parent, sibling or grandparent. The subsequent grief is multifaceted and each family member has different needs and resources. Through a systematic review of literature, we identified 24 studies between 1982 and 2021: they dealt with individual, family and couple experience when a SIDS occurs; in addition, some studies compared perinatal loss and neonatal loss with SIDS loss. Our results point out the need for an intervention that focuses on the needs of each family member and tailored around the specifics of SIDS loss rather than general grief.

## 1. Introduction

Sudden Infant Death Syndrome (SIDS) is “the sudden unexpected death of an apparently healthy infant less than one year of age that remains unexplained after a thorough case investigation, including performance of a full autopsy with ancillary testing, investigation of the site of death, and review of the clinical history” (Goldstein et al., 2019a, p. 626).

The Centers for Disease Control and Prevention refers to SUDI as a sudden and unexpected death of an infant occurred in the first year of life in which the cause was not apparent before investigation; these deaths often occur while the infant is asleep or in his or her sleep area. If no known cause of death can be determined despite several thorough investigations, SIDS may be used as a diagnosis of exclusion (Center of Disease Control and Prevention, 2021). This definition combines three cause-of-death categories in the International Classification of Diseases, 10th Revision (ICD-10): SIDS (code R95), unknown or unspecified causes (code R99), and accidental suffocation and strangulation in bed (code W75) [World Health Organization (WHO), 2019]. However, the debate over the labeling and classification of unexplained sudden infant deaths continues without a universally accepted standard procedure (Shapiro-Mendoza et al., 2021). The term SIDS has often been confused and criticized because it is not a well-defined diagnosis with precise pathognomonic features and its application can be highly subjective (Byard, 2018).

According to recent international comparisons, the SIDS rate in Europe is estimated at 25 cases per 100,000 live births. However, different definitions and study protocols have been noted, making relationships variable and international comparisons complex (de Visme et al., 2020). In Italy, the incidence is estimated to be approximately 250 new cases or 0.5‰, although no national registry is available (Ministry of Health, 2021). However, infant mortality has declined slightly in Western countries thanks to the spread of prevention campaigns (Moon, 2016). Nevertheless, some unexplained deaths remain, making it difficult to determine causes and to create a unified registry of surveillance data that makes them internationally comparable (Goldstein et al., 2019a).

The lack of a consistent and international methodology for evaluating cases can slow data collection and complicate the classification of SIDS deaths, which could be labeled differently depending on the jurisdiction in which they occur, which does not do justice to the death of the child itself (Byard et al., 2019). This fact underscores that it would be short-sighted to look for a single cause of sudden infant death syndrome. Rather, scientific evidence suggests the hypothesis that SIDS is the result of a combination of risk factors and pathophysiological responses that are different for each individual struggling with their own biological vulnerabilities and predispositions (Byard and Krous, 2003). Currently, the most relevant model in terms of risk factors is the “Triple Risk Model,” which states that SIDS risk is highest when the infant has all three identified factors: an individual vulnerability, a critical developmental period, and exposure to an exogenous stressor (Filiano and Kinney, 1994).

Although some medical and epidemiological issues remain unresolved, psychological research has begun to examine the consequences for families experiencing this painful event (Goldstein et al., 2019a). The loss of an infant to SIDS is a profound and tragic experience (Goldstein et al., 2018, 2019b,^2020^, ^2022^) and includes not only the death of the infant, but also the loss of an imagined and idealized family future and the fading of future expectations as parents, siblings, or grandparents. Grief in the family has many facets, and each family member grieves in different ways, has different needs and resources (Walsh and McGoldrick, 1991, 2013).

However, compared to perinatal and neonatal losses, SIDS occurs suddenly, remains unexplained, and is followed by exhausting medico-legal procedures for parents (Dyregrov and Matthiesen, 1987; Boyle et al., 1996), which suggests to us a more complicated grieving process (Goldstein et al., 2022). As observed in perinatal losses (Gandino et al., 2020), the death of a child from a sudden and unexplained cause can also have consequences for health care workers and professionals (Forster and Hafiz, 2015).

After the joy of the birth of their child, the family is confronted with the loss of light, as in an “eclipse” where a celestial body obscures the source of light, leaving the observer in an inevitable cone of shadow. The unexpected and unexplained loss of a baby is a particularly destabilizing event for the family (Byard, 2009) precisely because of the phenomenology with which it occurs and the short- and long-term consequences (Goldstein et al., 2019b). Upon discovery of death, parents find themselves at a “crime scene,” surrounded by police, coroners, and emergency responders (Byard, 2009). Often forgotten in the aseptic and rigid coronial procedures is an aspect of SIDS that is anything but unimportant: the grieving family (Goldstein et al., 2022).

According to our systemic relational perspective, death can be described as an event that affects the entire family; in fact, all individuals within the family system suffer from the loss at both the personal and relational levels (Walsh and McGoldrick, 1991, 2013). After a loss, family life is shaken to its foundations and must inevitably reorganize its structure and build a new one around the loss (Boss and Greenberg, 1984). For all family members, any death involves multiple losses: of the deceased person, of roles and relationships, of the family unit, of hopes and ideas about the future. To understand the nature of grief in the family, it is important to recognize that the individual and relational effects of loss operate simultaneously and are influenced by each other; in fact, individual grief is both matrix and product of change within the entire family system (Gilbert, 1996). Based on the assumption that grief is both a social and a familial process (Neimeyer et al., 2014), we hypothesize that SIDS may have psychological effects not only on the mother, but also on the father, couple, siblings, grandparents, and ultimately the family system.

This systematic review arose from the need to provide a qualitative synthesis of the psychological impact of SIDS not only at the personal level, but also on family and couple dynamics. A better understanding of what parents, siblings, and extended family experience is useful in providing forms of support or possibly interventions that address the needs and priorities of these individuals. The grief that follows the loss of a child to SIDS crashes into the lives of affected families like a bolt from the blue, and although there is a strong commitment to prevention campaigns worldwide, the etiology of sudden infant death syndrome is not yet clear. Therefore, until medical science provides the answers we all expect, it is of great interest to explore not only the risk factors for SIDS, but also to be prepared for what this event means at the couple and family level. This review should serve as a starting point for timely and up-to-date training of health professionals who should utilize the figure of the psychologist as the primary coordinator for family bereavement care.

The research questions that this systematic review aims to answer are: What are the consequences of SIDS at the personal and family level? What is the grieving process of the mother, father, couple, and other family members? The aim of this review is therefore to develop a better understanding of the difficulties faced by bereaved families after a fatality attributed to SIDS.

## 2. Materials and methods

### 2.1. Information sources and research strategies

The following systematic review was conducted in accordance with the Preferred Reporting Items for Systematic Reviews and Meta-Analyses (PRISMA) guidelines for searching, systematizing, and reporting systematic reviews (Moher et al., 2009; Page et al., 2021). The search was conducted from July 2021 to December 2021 and has no specific time interval. It was decided not to limit the search to a short time frame in order to include as many studies as possible. Each article was independently reviewed for eligibility by two individuals.

Studies were identified by querying online databases (ProQuest Psychology Journal, PsycARTICLES, PsycINFO), focusing on the psychological impact of SIDS at the family, personal, and couple levels. The combination of keywords in this first step was: (1) sudden infant death syndrome OR sids OR sudi OR suid OR sudden unexplained death in infants, AND (2) bereavement OR grief, AND (3) parent’ OR mother’ OR father’ OR sibling’.

### 2.2. Selection of articles

**FIGURE 1:**
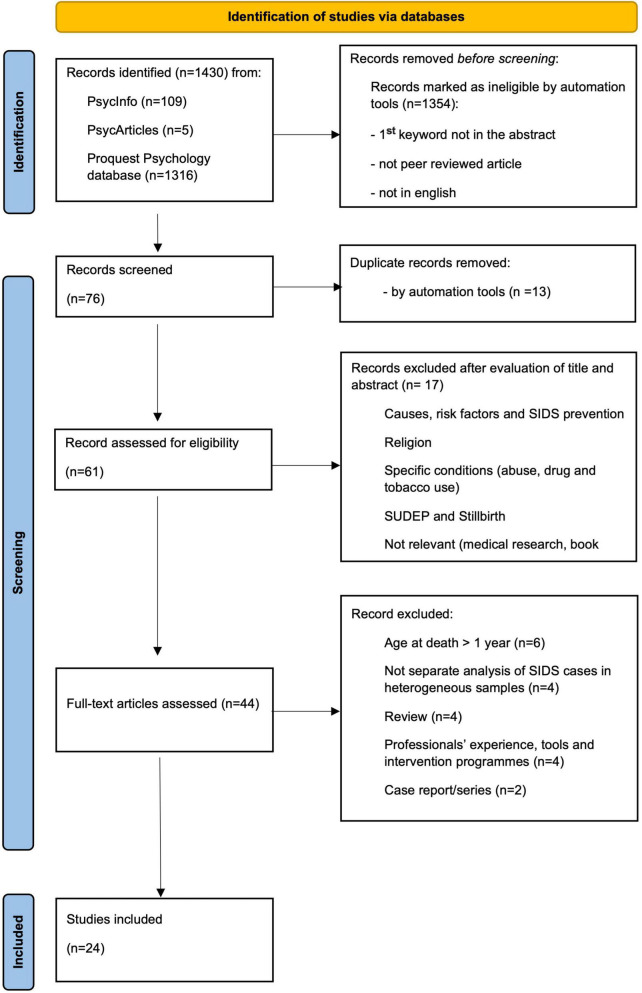
PRISMA flow diagram for literature search.

The database search yielded a total of 1,430 studies. This output was screened to select only those studies that met the defined objectives. The following criteria were used in the selection process: (1) the initial keyword search was limited to the abstract to ensure greater relevance to the topic under investigation, (2) they were peer-reviewed articles, and (3) they were published in English. Given our focus and our aim to conduct a comprehensive and complete analysis, we chose English-language publications because of the greater amount of international literature available. A total of 1354 studies were screened and 76 met the first stage criteria (Figure 1).

Subsequently, this number was further reduced to 61 after eliminating 15 duplicates. 13 records were automatically removed, while 2 duplicates were manually removed.

After duplicates were removed, the 61 selected studies were analyzed by title and abstract, resulting in a group of 44 articles. Of these, 17 articles were removed because they (1) addressed causes, risk factors, and SIDS prevention; were more concerned with religion than with the consequences of SIDS for family members; addressed specific conditions (abuse, drug, and tobacco use), SUDEP, and stillbirths; and, finally, addressed medical research or book reviews. Studies that addressed the search for medical causes of SIDS or risk and prevention factors were excluded because, although they were of great scientific relevance, they were not consistent with the objectives of the present study.

In the fourth and final selection phase, 44 full-text studies were assessed and reduced to 24, as 20 did not meet the inclusion criteria. Because SIDS occurs in the first year of life, it was decided to exclude all studies with samples in which death occurred after the first year of life (*n* = 6); to focus on a specific typology rather than a general category, studies whose sample included a broader range of perinatal/neonatal loss experiences were excluded without a separate analysis for SIDS cases (*n* = 4); literature reviews (*n* = 4) and case reports/series (*n* = 2) were excluded due to lack of new research data and difficulty of generalization. Studies addressing professionals, instruments, and intervention programs (*n* = 4) were considered ineligible because they did not meet the requirements of the research question. The remaining 24 articles-see Figure 1 for the PRISMA flow diagram-meet the inclusion criteria and were subjected to qualitative analysis to answer the research questions.

## 3. Results

The systematic analysis of the literature was carried out considering the objectives, the inclusion and exclusion method used and the results. The selection process is shown in Figure 1, while detailed information on each article can be found in Table 1.

**TABLE 1:** Summary table—studies about the psychological impact of SIDS on the family system.

References	Country	Methodology	Target population and sampl	Measures	Relevant results (for this review)	Strengths	Weaknesses
Williams and Nikolaisen (1982)	USA	Empirical study, quantitative study	Mothers and fathers *n* = 54 parents	*Ad hoc* questionnaire	Differences between partners were examined. Mothers described more emotional reactions in contrast to fathers; however, both considered themselves capable of expressing their feelings. fathers appeared to be less passive than mothers and more action oriented	Mothers’ and fathers’ responses were examined and compared separately	Small sample, limited geographic location, retrospective responses
Rubin (1984)	Israel	Empirical study, qualitative study	Mothers *n* = 15 women	In-depth interviews	Mothers who lost their infant to SIDS approximately four and a half years earlier still feel a lingering sense of responsibility and guilt for their child’s death. Adjustment is complicated by the lack of knowledge about the cause of death	Detailed exploration of topics	Small sample
Price et al. (1985)	USA	Empirical study, quantitative study	Mothers *n* = 73 women	*Ad hoc* questionnaire	The age of the child at the time of death appears to be correlated with maternal adjustment. More than half of the sample reported good marital adjustment and closeness to other children after the loss. Despite doubts and uncertainties, the vast majority of mothers indicated that they wanted to have another child	At the time of the article’s publication, it was considered an important source because it revealed new areas of research	Unclear variables explaining the difference between mothers with unsatisfactory adaptation and those with satisfactory adaptation
Dyregrov and Matthiesen (1987)	Norway	Empirical study, quantitative study	Mothers and fathers *n* = 117 parents	*Ad hoc* questionnaire, including: The Impact of Event Scale (IES) 20-item Goldberg General Health Questionnaire (GHQ-20) State-Trait Anxiety Inventory (STAI) Bodily Symptom Scale (BSS) - Beck Depression Inventory Short Form (BDI-SF)	Compared with the other types of infant loss, the SIDS category had higher mean scores on all measures, indicating greater stress after loss. Adjustment correlated with infant lifespan, with parents whose infant had lived longer experiencing greater difficulty	Stillbirth, neonatal and SIDS group’s responses were explored separately and compared	Retrospective responses: high probability that estimates are lower than actual prevalence of bereaved parents due to non-response rate; only mother’s questionnaire included questions about sibling reactions
Downey et al. (1990)	USA	Empirical study, quantitative study	Mothers and fathers *n* = 124 parents	Symptom check list-32 structured interview	Parents who blamed themselves or someone else for the death of their children were more distressed, whereas attributions to chance were unrelated to distress. The hypothesis that attributions influence subsequent adaptation is not supported	Relatively large, economically, and racially diverse sample; investigation on temporal changes in the attribution process	The interview timing could not detect changes in the dependent variables caused by independent variables
Powell (1991)	Ireland	Empirical study, quantitative study	Siblings *n* = 78 children (from *n* = 28 mothers’ and *n* = 23 fathers’ impressions).	Structured interview	Following SIDS, siblings show more attention-seeking behavior and regressions to earlier developmental stages. Sometimes internalizing behavior increases, while externalizing behavior is accentuated in others. Parents often find themselves unprepared to deal with children’s persistent curiosity about loss	Probability sampling; each parent’s responses were recorded separately; high level of agreement between partners	Children not directly involved
De Frain et al. (1992)	USA	Empirical study, mixed methods study	Grandparents *n* = 80 grandparents	*Ad hoc* questionnaire	Grandparents’ grief is unique. They could also feel anger due to the lack of explanations for SIDS and tend to create their own theories about their grandchild’s death. Even though they try to be strong, they admit that their own suffering also needs support	Qualitative data deepen quantitative findings	Small sample; the majority of the respondents were grandmother and on the maternal side; high probability that the estimates are lower than the real prevalence as sampling was done through support groups
Carroll and Shaefer (1993)	USA	Empirical study, quantitative study	Mothers and fathers 34 couples (*n* = 68 parents)	Structured interview SIDS Parent Coping Inventory (SPCI)	Both parents acknowledged their loss but coped with their grief in different ways. Communication and partner support were the predominant coping methods. Compared to mothers, fathers’ feelings are not expressed by crying or seeking support outside their partner relationship	High internal consistency	Small and self-selected sample; exclusion of bereaved parents who no longer live together after their loss
Ostfeld et al. (1993)	USA	Empirical study, quantitative study	Mothers *n* = 38 mothers	NJSRC Parent Questionnaire	Bereaved mothers experience a reduction in all symptoms compared to the early acute pain. Single mothers experience higher grief levels both immediately and 6 months after loss; they are more likely to become pregnant and to move elsewhere. Women who were pregnant or planning another pregnancy showed lower early grief scores	The Likert scale allows you to capture the nuances of the maternal experience and provide a specific focus on maternal needs. A focus on what the mother perceived as a resource at that moment	Small sample; high probability that the estimates are lower than the real prevalence as sampling was done through a resource centre; the aspects of the couple relationship that are limiting to a better resolution of maternal pain
Hutton and Bradley (1994)	Australia	Empirical study, quantitative study	Siblings 38 children (from *n* = 23 mothers’ impressions) Control group: 40 children (from *n* = 30 mothers’ impressions).	Child Behavior Checklist (CBCL) Semi-structured interviews	Siblings aged between 4 and 11 showed more behavioral problems than the control group, with no significant improvement from 7 to 18 months after loss. No prototypical bereaved child was identified, but significantly higher scores were found on the subscales concerning depression, aggression, social withdrawal, and sexual problems	Semi-structured interview helps introduce data that may be biased by mothers’ experiences; an attempt to help health professionals dealing with siblings’ reactions after SIDS; non-participation of fathers in survey may be a question and a starting point to better understand the process of paternal grief and its expression; relatively representative sample	High probability that the estimates are lower than the real prevalence; elevation of behavior problems associated with the cot death of a sibling could be non-specific; children not directly involved; only mothers’ responses were analyzed
Powell (1995)	Ireland	Empirical study, quantitative study	Nuclear family *n* = 69 parents	Structured interview	Parents who carried on another pregnancy pointed out that it was a common coping strategy during the first year after loss. The grieving process was not inevitably inhibited, indeed for many fathers the subsequent pregnancy was related to the perceived acceptance of SIDS	Probability sampling; each parent’s responses were recorded separately	Small sample
Boyle et al. (1996)	Australia	Empirical study, quantitative study	Mothers *n* = 194 mothers Control group: *n* = 203 mothers.	Delusions Symptoms States Inventory/states of Anxiety and Depression (DSSI/sAD)	Mothers in the SIDS group show a higher risk of psychological distress than the control group, with higher rates of anxiety and depression in the first months after loss, slowly decreasing over time. SIDS loss appears to increase the risk for parents to develop anxiety disorders and depression, rather than stillbirth or neonatal death	Relatively large sample; comparison group of non- bereaved families; longitudinal study; stillbirth, neonatal and SIDS group’s responses were explored separately	No pre-loss measures of mental health available; probable sample loss effect
Thuen and Schlytter (1996)	Norway	Empirical study, quantitative study	Mothers and fathers *n* = 251 parents Control group: *n* = 973 parents.	Symptom Check List-90	Both parents experience their loss in different ways, mainly from 2 to 5 years later. Many mothers seem to show distress symptoms beyond 2 years after loss. Child’s life length had no effect on parental adaptation in the present study. Individual adaptation improved over time, and it correlated between partners	Relatively large sample; comparison group of normal population	High probability that the estimates are lower than the real prevalence as sampling was done through a society which provides support and information
Thuen (1997)	Norway	Empirical study, quantitative study	Mothers and fathers *n* = 251 parents	*Ad hoc* questionnaire, including: Symptom CheckList-90 Bradburn’s affect-balance scale	No significant gender differences were found in the amount of instrumental, emotional, and informational support received by couples experiencing SIDS. Instrumental and informational support are more strongly correlated with psychological adjustment (emotional support may also be included within them)	Relatively large sample	High probability that the estimates are lower than the real prevalence as sampling was done through a society which provides support and information; low response-rate
Väisänen (1998)	Finland	Empirical study, qualitative study	Mothers, fathers, siblings, and grandparents *n* = 56 individuals	Interviews and focus group	Perinatal death, neonatal death and SIDS are traumatic experiences for the family, also presenting some post-traumatic symptoms in mothers. Family bereavement is a multi-faceted process and parents try to recreate the child, in their mind, psychologically or spiritually	Detailed exploration of topics	Small sample; double orientation approach (therapist and researcher)
Dyregrov and Dyregrov (1999)	Norway	Empirical study, Mixed methods study	Mothers and fathers *n* = 25 parents	QQuestionnaire Impact of Event Scale (IES) 20-item GHQ State Trait Anxiety Inventory-State version (STAI-X1) Bodily Symptom Scale (BSS) Beck Depression Inventory Short Form (BDI-SF) Semi-structured, in-depth interviews	Parents who have experienced SIDS have similar mean scores 12 to 15 years after loss, with most fathers still at risk of psychological distress. Interviews show a strong fear of something happening to other children, especially in the post loss period and during a subsequent pregnancy	Qualitative data deepen quantitative findings	Small sample
Irizarry and Willard (1999)	Australia	Empirical study, quantitative study	Mothers and fathers *n* = 61 parents	*Ad hoc* questionnaire	Following the loss, women have more intense reactions (insomnia, anxiety, lack of concentration and motivation). More women than men wanted a subsequent pregnancy soon; men reported an increase in the desire for sexual activity following the death while women reported a decrease	Mothers and fathers’ responses were explored separately; respondents completed in respect of him/herself and in respect of his/her partner	Small sample
Edwards et al. (2009)	New Zeland	Empirical study, qualitative study	Fathers *n* = 9 fathers	In-depth interviews	According to Maori fathers’ narratives, being treated like criminals during the investigation it’s a significant stressor. Men pointed out the importance of staying busy with their own work and routines. Men are not well supported by services and social isolation and loneliness are common feelings	Detailed exploration of topic	Small sample; specific cultural context
Finlay and Krueger (2011)	USA	Empirical study, qualitative study	Mothers *n* = 20 websites	Textual analysis	Creating and developing memorial websites helps to cope with grief, fulfilling the need for self-expression, helping to rebuild identity, and giving meaning to loss	Detailed exploration of online mourning process	Small sample; social networks excluded
Garstang et al. (2016)	UK	Empirical study, Mixed methods study	Mothers and fathers 21 families (*n* = 34 parents) *n* = 27 professionals.	Hospital Anxiety and Depression Scale (HADS) Questionnaires In-depth interviews	Parents who have experienced SUDI or SIDS have a strong need to know the reason for their child’s death. According to this study, sharing detailed information about baby’s death is a real parent’ wish and it’s not related to self-blame	Qualitative data deepen quantitative findings; data from parental interviews, professional interviews, questionnaires, and case records; SUDI and SIDS group’s responses were explored separately	Small sample
Goldstein et al. (2018)	USA – South Africa	Empirical study, quantitative study	Mothers *n* = 356 mothers.	Parental Bereavement Questionnaire (PBQ)	SIDS loss is associated with high levels of grief in the child’s parents who are at a higher risk of developing *Prolonged grief disorder*, between 1 and 3 years after loss. The most persistent symptoms are role confusion, anger, and diminished trust	Large sample	Selection bias; determination of PGD using a survey; limited statistical power
Goldstein et al. (2019b)	USA – South Africa	Empirical study, quantitative study	Mothers *n* = 50 mothers Control Group: *n* = 124 mothers	Spielberger State-Trait Anxiety Inventories (STAI-T and STAI-S) Edinburgh Postnatal Depression Scale (EPDS) Timeline Follow-Back (TLFB) Parental Bereavement Questionnaire (PBQ)	Bereaved mother’s response to SIDS is significantly determined even before the loss occurs; vulnerability factors have a cumulative effect, even if limited in time	Comparison group of non-bereaved mothers; prospective data collection before the infant’s death	Small sample; limited statistical power
Goldstein et al. (2020)	USA – South Africa	Empirical study, quantitative study	Mothers *n* = 294 mothers	*Ad hoc* questionnaire, including: Parental Bereavement Questionnaire (PBQ)	According to this study, transitional objects have a potential therapeutic role for bereaved mothers. This research also illustrates Prolonged Grief Disorder as a disorder of attachment. Rather than benefiting from the restorative aspects, mothers with PGD experience distress with transitional objects which impede access to positive aspects of the relationship	Large sample	Selection bias; determination of PGD using a survey; lack of data on the attachment style in the infant-mother relationship; no data on paternal behavior; increased risk of type I error due to the number of statistical tests
Plews-Ogan et al. (2021)	USA	Empirical study, qualitative study	Mothers and father *n* = 53 parents	Semi-structured interviews	Loss involves significant changes in the parental role, from the physical to the emotional sphere, to a new meaning construction. Parents described the effects of their infant’s death on how they were a parent to their other children, suggesting an intergenerational transmission of this family event	Detailed exploration of topics	Small sample; possible selection bias; high probability that the estimates are lower than the real prevalence as sampling was done through a hospital program

The selected studies were conducted in different parts of the world: United States (*n* = 11), Israel (*n* = 1), Norway (*n* = 4), Finland (*n* = 1), Ireland (*n* = 2), United Kingdom (*n* = 1), Australia (*n* = 3), New Zealand (*n* = 1), and South Africa (*n* = 3).

They cover a period of four decades, from 1982 to 2021, with: a greater frequency in the 1990s (*n* = 13), followed by the period from 2010 to 2020 (*n* = 4), the 1980s (*n* = 4), from 2020 to the present (*n* = 2), and the period from 2000 to 2010 (*n* = 1).

Sixteen studies used quantitative techniques, five used qualitative methods, and three used a mixed methodology.

### 3.1. Data extractions

As can be seen from Table 1, the review of the literature reveals that six *ad hoc* questionnaires have been used in quantitative studies (Williams and Nikolaisen, 1982; Price et al., 1985; Dyregrov and Matthiesen, 1987; Thuen, 1997; Irizarry and Willard, 1999; Goldstein et al., 2020). Of these six studies, three also used other instruments, such as the Impact of Event Scale (IES), the 20-item Goldberg General Health Questionnaire (GHQ-20), the State-Trait Anxiety Inventory (STAI), the Bodily Symptom Scale (BSS), the Beck Depression Inventory Short Form (BDI-SF) (Dyregrov and Matthiesen, 1987), the Symptom ChackList-90 and Bradburn’s Affect-Balance Scale (Thuen, 1997), and the Parental Bereavement Questionnaire (PBQ) (Goldstein et al., 2020). Four studies used structured interviews (Downey et al., 1990; Powell, 1991, 1995; Carroll and Shaefer, 1993), and of these four studies, two included additional instruments such as the Symptom Check List-32 (Downey et al., 1990) and the SIDS Parent Coping Inventory (SPCI) (Carroll and Shaefer, 1993). One study used semi-structured interviews and also incorporated the Child Behaviour Check List (CBCL) (Hutton and Bradley, 1994). Of the original sixteen quantitative studies, five used only scales such as: NJSRC Parent Questionnaire (Ostfeld et al., 1993); Delusions Symptoms States Inventory/States of Anxiety and Depression (DSSI/sAD), (Boyle et al., 1996); Symptom Check List-90 (Thuen and Schlytter, 1996); Parental Bereavement Questionnaire (PBQ), (Goldstein et al., 2018); Spielberger State-Trait Anxiety Inventories (STAI-T and STAI-S), Edinburg Postnatal Depression Scale (EPDS), Timeline Follow-Back (TLFB), PBQ (Goldstein et al., 2019b). Of the five qualitative studies, two used in-depth interviews (Rubin, 1984; Edwards et al., 2009), one study used interviews and focus groups (Väisänen, 1998), one study conducted text analysis (Finlay and Krueger, 2011), and one study used semi-structured interviews (Plews-Ogan et al., 2021). Finally, of the three mixed-method studies, one used an *ad hoc* questionnaire (De Frain et al., 1992), one used a questionnaire with scales such as the IES, the 20-item GHQ, and the STAI State Version (STAI-X1) (Dyregrov and Dyregrov, 1999), and one used a questionnaire, in-depth interviews, and the Hospital Anxiety and Depression Scale (HADS) (Garstang et al., 2016).

### 3.2. Analysis of the samples

The sample of each study refers exclusively to SIDS cases, with the exception of a minority that uses a heterogeneous sample that includes perinatal, neonatal, and SUDI deaths (Dyregrov and Matthiesen, 1987; Boyle et al., 1996; Väisänen, 1998; Garstang et al., 2016). In addition, the sample of 24 studies includes 9 to 365 participants, of which only 4 had a control group (Hutton and Bradley, 1994; Boyle et al., 1996; Thuen and Schlytter, 1996; Goldstein et al., 2019b). All studies analyzed a sample of subjects, with the exception of one study that focused on evaluating 20 websites dedicated to victims of SIDS, but from which we conventionally derived the activity of 20 subjects (Finlay and Krueger, 2011). Finally, data from studies of siblings come from parental perceptions, particularly maternal perceptions, and therefore indirectly examine siblings’ experiences (Powell, 1991; Hutton and Bradley, 1994).

### 3.3. Family members affected

The selected articles address the experiences of those affected by a family tragedy due to SIDS. We found that eight of them focus on the experiences of mothers (Rubin, 1984; Price et al., 1985; Ostfeld et al., 1993; Boyle et al., 1996; Finlay and Krueger, 2011; Goldstein et al., 2018, 2019b,^2020^), one on the paternal experience (Edwards et al., 2009), 10 on the couple (Williams and Nikolaisen, 1982; Dyregrov and Matthiesen, 1987; Downey et al., 1990; Carroll and Shaefer, 1993; Thuen and Schlytter, 1996; Thuen, 1997; Dyregrov and Dyregrov, 1999; Irizarry and Willard, 1999; Garstang et al., 2016; Plews-Ogan et al., 2021), two on sibling experiences (Powell, 1991; Hutton and Bradley, 1994), one on the family unit consisting of mother, father, and children (Powell, 1995), one on grandparents (De Frain et al., 1992), and one on the extended family consisting of mother, father, children, and grandparents (Väisänen, 1998).

### 3.4. Thematic features

The 24 studies included in the review provide an overview of the SIDS event and illustrate in its complexity the many aspects associated with the sudden death of a newborn and the associated impact at all levels of the family. Williams and Nikolaisen (1982), Carroll and Shaefer (1993), Thuen and Schlytter (1996), and Irizarry and Willard (1999) focused on the gender differences that exist between parents due to SIDS, examining the adjustment strategies used by each partner and the different reactions that occur even after a long time. This last aspect is addressed by Rubin (1984) and by Dyregrov and Dyregrov (1999), who examined the difficult adjustment to such a painful and traumatic loss that does not seem to heal completely even with time, leaving parents with the painful feeling of still having to deal with an unexplained death. In such situations, there is a risk of developing a persistent and complicated bereavement disorder, for which a higher risk of diagnosis was found in the population that experienced a SIDS death in the family (Goldstein et al., 2018), although this is also likely related in part to pre-loss risk factors (Goldstein et al., 2019b).

Several studies (Price et al., 1985; Downey et al., 1990; Ostfeld et al., 1993; Väisänen, 1998; Plews-Ogan et al., 2021) have focused on the issues associated with parental and family loss, ranging from sharing the pain, attributive concerns, adjustment to marriage, impact on desire for more children, and the complex nature of the recovery process, to reinvesting energies to move forward and live more serenely with the reality of loss (Price et al., 1985; Downey et al., 1990; Ostfeld et al., 1993; Väisänen, 1998; Plews-Ogan et al., 2021). The desire to chase life moving forward rather than remaining immobile is confirmed by Powell (1995), who showed how a child in the year following the loss did not inhibit the grieving process but enhanced it by restoring meaning and planning to a life emptied by SIDS. However, the siblings of children who have been victims of SIDS need adequate support and an environment that often protects them from any manifestation of discouragement and sadness and that might set the stage for behavioral and personality change (Powell, 1991; Hutton and Bradley, 1994).

Grandparents were also considered in this study. For example, De Frain et al. (1992) study examined their experiences, feelings of guilt, tendency to relive the death of their nephew, but also the strength of their marriage and ability to rise again thanks to faith. Thuen (1997) shows that parents can access various forms of support, such as instrumental, emotional, and informational support. They may cope with the painful grieving process through the use of memorial websites (Finlay and Krueger, 2011) or through the use of transitional objects, although this experience of contact seems to be influenced by the intensity and severity of the pain experienced (Goldstein et al., 2020).

Parents also seek to authentically share detailed information about the diagnosis, which facilitates a comprehensive explanation of the death by professionals (Garstang et al., 2016).

The review also includes studies comparing SIDS to stillbirth and neonatal death, highlighting the differences in grief and mental health outcomes for those affected in their various facets (Dyregrov and Matthiesen, 1987; Boyle et al., 1996). In addition, one study has illustrated the unique situation of an indigenous people, the Māori, who are widespread mainly in New Zealand and have a high risk for SIDS deaths (Edwards et al., 2009).

In conclusion, the review provided satisfactory results at the substantive level, indicating that the topic deserves clinical attention and inspiration for further research, not only in the medical field but also in the psychological field.

### 3.5. Strengths and weaknesses of the analyzed literature

The strengths and weaknesses of the studies examined in the systematic review relate to both the methods used to conduct the research and the themes that guided the investigation. As can be seen from the Table 1, from the aspects of the strength of the literature reviewed, it can be noted that some studies have conducted a detailed investigation of the topic under investigation (Rubin, 1984; Väisänen, 1998; Edwards et al., 2009; Finlay and Krueger, 2011; Plews-Ogan et al., 2021); others have a large sample (Goldstein et al., 2018, 2020), a relatively large sample (Downey et al., 1990; Hutton and Bradley, 1994; Boyle et al., 1996; Thuen and Schlytter, 1996; Thuen, 1997), or a probability sample (Powell, 1991, 1995).

The Price et al. (1985) study provides an overview of the major issues and offers study perspectives for the future. Some studies have examined mothers’ and fathers’ responses separately (Williams and Nikolaisen, 1982; Powell, 1991, 1995; Irizarry and Willard, 1999), and in some cases a high degree of agreement between partners has been found (Powell, 1991). Fathers have participated less in research than mothers (Hutton and Bradley, 1994). We believe that it could be interesting to understand the clinical motivation behind this mechanism and lead research to new horizons. Some research shows that qualitative data deepen quantitative findings (De Frain et al., 1992; Dyregrov and Dyregrov, 1999; Garstang et al., 2016). Finally, some studies have: conducted perspective-taking data collection prior to infant death (Goldstein et al., 2019b), examined responses separately by death category when examining differences (Dyregrov and Matthiesen, 1987; Boyle et al., 1996; Garstang et al., 2016), placed a particular emphasis on the nuances of maternal experience (Ostfeld et al., 1993), and when method analysis identified bias, the presence of semi-structured interviews likely helped reduce the bias effect (Hutton and Bradley, 1994).

The weaknesses of the studies reviewed in some cases show weaknesses in the number or quality of the sample of the population studied (Rubin, 1981; Williams and Nikolaisen, 1982; De Frain et al., 1992; Carroll and Shaefer, 1993; Ostfeld et al., 1993; Powell, 1995; Väisänen, 1998; Dyregrov and Dyregrov, 1999; Irizarry and Willard, 1999; Edwards et al., 2009; Finlay and Krueger, 2011; Goldstein et al., 2019b; Plews-Ogan et al., 2021). In some circumstances, some studies show a high probability that estimates are lower than actual prevalence (Dyregrov and Matthiesen, 1987; Ostfeld et al., 1993; Hutton and Bradley, 1994; Thuen and Schlytter, 1996; Thuen, 1997); however, other studies haven’t clarified the differences in the results obtained and the variables associated with this variation (Price et al., 1985); the exclusive presence of maternal responses when children are studied (Dyregrov and Matthiesen, 1987; Powell, 1991); the lack of pre-loss measures of mental health available (Boyle et al., 1996); the presence of selection bias; and the determination of PGD using a survey (Goldstein et al., 2018, 2020).

## 4. Discussion

This literature review identified 24 studies on the psychological impact of SIDS on the family system. This literature review highlighted the basic characteristics of SIDS: first, the psychological impact on mothers, fathers, couples, siblings, grandparents, and the entire family system; and second, the differences between SIDS and perinatal and neonatal loss.

### 4.1. Mothers

Ostfeld et al. (1993) found that mothers who had experienced SIDS recalled the initial acute grief, which was characterized by intense symptoms of sadness, difficulty concentrating, restlessness, sleep disturbances, and anger. In the six months following the loss, although sadness remained the most intense symptom, followed by anger, the others decreased markedly, giving way to anxiety, discomfort with the babies, and guilt, symptoms attributed to the cognitive rather than the somatic aspects of grief (Ostfeld et al., 1993).

Price et al. (1985) also emphasized the presence of a persistent experience of sadness and depression as major symptoms and decreasing levels of restlessness, anxiety, sleep disturbance, difficulty concentrating, discomfort with other pregnant women, less energy, less interest in social activities, loss of appetite, guilt, and work difficulties. Consistent with the psychological effects described in previous studies, Goldstein et al. (2018) highlighted interesting aspects, such as the presence of symptoms that decrease over time and symptoms that remain relatively stable, such as role confusion, anger, and distrust; these data enabled the finding that Prolonged Grief Disorder rate is 57.1% one year after loss and 41.3% after three years (Goldstein et al., 2018).

Given these findings and the shared risk factors between SIDS grief and PGD, Goldstein et al. (2019b) found an interesting association between individual pre-loss vulnerability factors and the development of PGD after SIDS, with the influence decreasing with time after loss. This study, chronologically among the most recent to be included in this review, highlights key factors that predict the characteristics of individuals who do and do not suffer from PGD (Goldstein et al., 2019b). Examination of the evolution of each risk factor in the 30 months after loss showed different trajectories for PGD: Maternal age greater than 26 years predicted higher rates of PGD symptoms; preloss depressive symptoms had an impact up to 2 years after loss; preloss anxious symptoms predicted higher levels of acute grief but not of persistent and complicated grief disorder, which cannot be diagnosed until at least 6 months after loss; women who had experienced previous losses had lower but non-significant rates of PGD than women who had not experienced a loss. In addition, the presence of other living children proved to be a risk factor, as these mothers showed a decreasing risk of PGD only up to two years after loss, followed by an increase likely due to comparison with the deceased child during bonding with the next child or upon reaching the age of the deceased child; higher alcohol consumption was consistently positively associated with PGD (Goldstein et al., 2019b). The authors strongly emphasized the risk posed by the simultaneous presence of multiple factors: more than two factors significantly predicted risk in the year after death, while four factors significantly predicted risk 2 years after the child’s death.

Interviews conducted in Rubin (1984) study confirmed the distressing nature of the loss, which was primarily due to the timing of the death, i.e., in the middle of the attachment process (Rubin, 1984). Early symptoms such as depression, anxiety, and helplessness subside, whereas guilt symptoms continue to emerge years later, independent of the other symptoms. This study suggests that guilt is the main feature of SIDS tragedy: According to Rubin (1984) and Plews-Ogan et al. (2021), deep despair is triggered by the idea of not being able to protect the child, because the inability to understand the causes of death is a factor strongly associated with maternal adjustment problems. Following Parkes (1970), Rubin (1981, 1984), Finlay and Krueger (2011) and Goldstein et al. (2020) studies confirm the need to continue an activity that involves the “presence” of the deceased child in order to process the loss. Finlay and Krueger (2011) qualitative analysis examined 20 memorial websites, photos, poems, memories, and spaces for self-expression shared in personal language, in a non-professional manner, and without adherence to precise esthetic standards. These features make them particularly authentic and attest to the intention to create a sense of community, to share grief despite taboos, and thus to begin a process of healing and reconstruction of identity shattered by loss. The power of these places, also highlighted by the authors, lies in their constant accessibility, not determined by opening and closing times, without limits of permanence (Finlay and Krueger, 2011); moreover, in the work they do on two fronts, grief and recovery, in a dynamic process of oscillation, as described in the “Dual Process Model of Coping” (Stroebe and Schut, 1999). Sometimes there is a need to face the loss of a loved one and relive time spent together through photographs, special places, and music, but at other times people feel the need for a break to gradually return to life (Finlay and Krueger, 2011).

The presence of objects with strong adaptive potential has also been suggested: they have been referred to as transitional objects of grief (Goldstein et al., 2020). The association between Prolonged Grief Disorder (PDG) and feelings of distress in dealing with the object is clear and is also characterized by overwhelm in coping with the loss and lower frequency of visits, in contrast to the mothers who had experienced relief and comfort from the object (although the frequency did not differ significantly between the two groups). PDG is not directly related to avoidance behavior, but only when the use of the object produces stress in the presence of indicators of PDG. Clearly, these mothers not only have limited abilities to use the adaptive potential of transitional objects of grief, but their natural adjustment to loss is actually complicated lacking the oscillation necessary for a successful outcome (Goldstein et al., 2020).

### 4.2. Fathers

From the literature review, only one study emerged that focused exclusively on the father figure; however, given the reference sample, it cannot be considered representative of the population.

Programmes focused on prevention and reducing modifiable risk factors have led to a decrease in SIDS rates in New Zealand, although not significantly in the indigenous population (McManus et al., 2010). However, the most recent data available show that SUDI rates for Mâori and Pacific ethnic groups were significantly higher than for children of all ethnic groups in New Zealand between 2014 and 2018 (Ministry of Health, 2021). A thematic analysis of in-depth interviews by Edwards et al. (2009) identified three main themes underlying the interviews: stressors related to the timing of death, personal coping mechanisms, and sources of support (Edwards et al., 2009). Bereaved Māori fathers are not well supported by services: It is difficult to find someone who can help them cope with the loss of their SIDS child; instead, they try to find support and strength in their other children, but social isolation and loneliness are common feelings. Health services are currently tailored to the needs of women and therefore need to be designed to allow men to express their grief as well. The authors suggest activity-based support (Edwards et al., 2009).

Based on the findings, it could be noted that there is little literature on the figure of the father at the international level; moreover, in research on the grief experience of family members after a childhood loss, the mother’s experience has been much studied, to the detriment of the fathers’ experience (Morris et al., 2019). Therefore, further thematic knowledge is needed to better understand the experience of grieving fathers so that the life of the family as a whole is not lost after a loss.

### 4.3. Couples

SIDS results in a sudden change in the roles and responsibilities of both partners, which differ in terms of gender-specific grieving processes (Plews-Ogan et al., 2021): men’s problem-solving ability is more action-oriented than women’s (Williams and Nikolaisen, 1982).

The study by Plews-Ogan et al. (2021) provided a considerable amount of data on changes in parenting and highlighted the main difficulties related to maintaining a positive self-image as a parent. The literature clearly shows that the sudden and unexpected death of a child seriously challenges a parent’s ability to provide basic functions such as safety and security; in this way, parents become aware of their own limitations as they are unable to ensure their children’s survival at all costs and feel their role is severely compromised by this vulnerability (Osterweis, 1984; Duncan and Byard, 2018).

Studies examining gender differences in couples (Williams and Nikolaisen, 1982; Plews-Ogan et al., 2021) have highlighted aspects of closeness, but also those of possible misunderstanding (Williams and Nikolaisen, 1982; Carroll and Shaefer, 1993; Irizarry and Willard, 1999). On the one hand, women’s feelings seem to show higher intensity and emotionality (Williams and Nikolaisen, 1982; Irizarry and Willard, 1999), but on the other hand, no differences in the ability to express their feelings were found. There were clear and significant differences in the partners’ approach to problem solving, with men’s problem-solving ability being more action-oriented than women’s (Williams and Nikolaisen, 1982). In both Schwab (1990) and Carroll and Shaefer (1993) studies, more coping mechanisms were used by mothers than by fathers (Schwab, 1990; Carroll and Shaefer, 1993).

Irizarry and Willard (1999) found significant differences in two main ways: first, women’s desire for pregnancy as soon as possible was often stronger than that of fathers, who showed a restricted desire related to fear of having another child; second, the need for sexual intimacy was increased in half of the male sample and decreased in more than half of the female sample. This is an aspect that may represent an area of psychological distress between partners, especially as it is strongly related to pregnancy (Irizarry and Willard, 1999). In contrast to studies on Penumbra Baby (Reid, 2007), Replacement child syndrome (Cain and Cain, 1964), and Vulnerable child syndrome (Green and Solnit, 1964) studies, Powell (1995) study emphasizes the benefits of subsequent pregnancy after SIDS loss. In fact, a low percentage-or lack thereof-of idealization by parents, the importance placed on subsequent children, and the absence of an overprotective attitude were observed (Powell, 1995). One of the most commonly cited grief coping strategies is social support, which is used to a significant extent by women but only to a small extent by couples (Carroll and Shaefer, 1993). Finally, studies show that for the parent couple, the presence of other living children is a protective factor and resource (Carroll and Shaefer, 1993; Irizarry and Willard, 1999). People sought more comfort from their partners, but while women were more likely to seek support from others, men seemed to seek more support within the household (Irizarry and Willard, 1999).

### 4.4. Siblings

The few studies that are available on siblings show that after the SIDS event, internalizing (sadness, social withdrawal, insecurity, insomnia, nightmares) and externalizing problems (aggressive and attention-seeking behavior) increase and peak about three months after the loss (Powell, 1991; Hutton and Bradley, 1994). However, the lack of studies directly involving children may lead to uncertainty in the results, which may be biased by parents’ perceptions; therefore, it may be useful to involve them directly in studies designed specifically for them. According to these findings, parents have great difficulty providing explanations for the death of their little brother or sister (Powell, 1991; Hutton and Bradley, 1994). Therefore, it is necessary to promote professional support to deal with the different types of children’s grief and to fill the gap with deeper knowledge. Children who lose a sibling are not only deprived of a playmate, but also temporarily deprived of parental support and attention, experiencing potential trauma on two fronts (Hogan and DeSantis, 1994; Packman et al., 2006; Avelin et al., 2014).

### 4.5. Grandparents

Studies that have examined the experiences of grandparents after the loss of their grandchild have highlighted one particular feature, the dual nature of the grief experience. The grief relates both to the grandchild because of his or her death and to the grandparents’ own child because of the difficulties he or she faces (Rando, 1986; Gerner, 1990; Reed, 2000). The US study by De Frain et al. (1992) is the only study in the review that focuses exclusively on grandparents’ experiences with SIDS. The questionnaire they were given was designed to explore not only how the loss changed their lives, but also how they processed it and used it to cope with grief (De Frain et al., 1992). Thanks to the participation of 80 grandparents who had lost their grandchildren between two months and 12 years before the study, it was possible to identify some crucial aspects. After the inconclusive results of the autopsy, many grandparents are not able to satisfy their constant need for explanations and put forward some theories about the death of their grandchild, ranging from more medical to religious theories. It was also found that 29% of respondents blame parents for their child’s death, citing their own inability to understand the symptoms and their own inadequacy (De Frain et al., 1992). Some issues should also be considered by mental health professionals: 1% of this sample experienced domestic violence after the loss, 4% contemplated suicide, and 6% reported an increase in alcohol and drug use in the family. Although they reported being strengthened by this event, grandparents recognized that they needed support both immediately and afterward; indeed, 60% advocated support groups for parents and grandparents (De Frain et al., 1992). As documented by Nehari and colleagues, there is a risk of feeling isolated and having no space to express their grief (Nehari et al., 2007).

### 4.6. Guilt

Although not always thoroughly studied and researched, one of the recurring themes in the background of SIDS bereavement is the feeling of guilt due to the lack of a clear medical explanation, which fuels the family’s ongoing search for a cause (Raphael, 1983). Following a previous study on the needs of bereaved parents (Garstang et al., 2014) and another study included in this review Thuen (1997), Garstang et al. (2016) emphasize the strong need of parents to talk to health care professionals about the causes of their baby’s death, in order to understand the reason and finally feel relieved (Garstang et al., 2016). The lack of a clear explanation after long periods of time not only encouraged cause-seeking behavior, but in some cases led parents to feel that the reason for their baby’s death was withheld from them (Covington and Theut, 1993), which could jeopardize their already fragile mental health. In the study by Garstang et al. (2016), four themes related to guilt were identified: self-blame, blaming others, feeling guilty, and blaming no one. Although not explicitly explored in the interviews, feelings of guilt were mentioned by parents; grieving mothers most frequently reported self-blame (after both SUDI and SIDS), but it was not related to the cause of death, modifiable risk factors, or parents’ understanding of them, supporting the hypothesis that it may be a typical aspect of grief after the death of an infant. Because there is no reason to believe that self-blame is related to the factors listed, there is also no reason to discourage partners from sharing information about the infant’s death with health care professionals (Garstang et al., 2016). There is no research on the topic of shame. In a paper examining emotions following perinatal loss from a transcultural perspective, we hypothesized that women in collectivistic societies that promote an interdependent self are more likely to experience shame, whereas in individualistic societies that promote an independent self, they are more likely to experience guilt (Provera and Gandino, 2022).

However, it is important to consider these data for intervention purposes, especially after the worsening of some risk factors due to the COVID-19 pandemic, particularly in women for anxiety disorders and major depression (COVID-19 Mental Disorders Collaborators, 2021).

### 4.7. Comparison between SIDS, stillbirth and neonatal death

Dyregrov and Matthiesen (1987) and Boyle et al. (1996) agree that the loss of a SIDS infant increases the risk for parents to develop anxiety symptoms compared with stillbirth and neonatal death. The first study showed that parents who had experienced SIDS death between 1 and 4 years earlier had a statistically significant difference in anxiety scores compared to the other two groups: Very high anxiety scores were observed in 69% of cases, compared with 27% for parents who had experienced the neonatal death and 15% for stillbirth (Dyregrov and Matthiesen, 1987). The second study examined the impact of the infant’s death at 2, 8, 15, and 30 months after the loss and found higher anxiety scores and depressive symptoms in the SIDS sample at all time intervals. In contrast, mothers who were in the newborn and stillbirth group had lower scores at the last interval that were similar to those of the non-mourning control group; furthermore, anxiety symptoms in the SIDS group decreased slowly over time, with approximately 22% of mothers having anxiety symptoms at 30 months (Boyle et al., 1996).

In addition to their findings on anxiety disorders, Dyregrov and Matthiesen (1987) showed a statistically significant difference in the distress of newborn death in terms of anger, self-blame, agitation, and sleep disturbance, as well as differences from stillbirth in terms of higher labor intensity and intrusive thoughts in both groups. In addition, the SIDS sample showed a significant difference in recovery after loss compared with the other two groups. Regarding the relationship between adjustment and sudden death, the effect of suddenness on bereavement outcomes could not be confirmed. However, it has been suggested that the particularly traumatic and destabilizing circumstances in the families of SIDS cases may explain the differences between the SIDS group and the other two groups (Dyregrov and Matthiesen, 1987).

The authors also pointed to a possible positive effect of pregnancies after the loss and a correlation between the length of the child’s life and the parents’ adjustment, but, in contrast to Price et al. (1985), found that a longer time spent with the child negatively affected the parents’ grief response (Dyregrov and Matthiesen, 1987).

## 5. Conclusion

Consistent with our expectations, this analysis shows that SIDS is a tragic event that has psychological consequences at the individual, couple, and family levels. In this sense, the unexplained and sudden loss of a child in the first year of life can have profound effects on the entire family system: Indeed, each member of the family unit may suffer from the loss at both the individual and relational levels.

According to the systemic-relational paradigm, the life of a family is characterized by some crucial steps in family life, and birth is precisely an event that can change the balance and bring about changes in family relationships. Psychological perinatality is a dynamic mental process that begins at conception and continues throughout the first year of life. During this time, the psychological and relational structure of the couple and family changes, expectations of the child are raised, and eventually the couple finds a new equilibrium, moving from the marital dyad to the parental triad. In a joyful and happy moment for the couple and the family, the SIDS comes in an inexplicable way, the whole family system is covered by a cone of shadow and everyone has to deal with the consequences of the death in a different way. Unlike stillbirths, in SIDS the couple and family have been able to get to know the child and form an attachment relationship (Rubin, 1984). Instead, in SIDS, unlike neonatal losses (Dyregrov and Matthiesen, 1987), guilt and self-blame (Garstang et al., 2016) typically emerge as a major feature of the tragedy (Rubin, 1984) and complicate adjustment to the loss (Plews-Ogan et al., 2021).

According to a constructionist approach, grief is an inherently social process, and after a loss, the search for meaning encompasses the entire family and community context in which meanings of life and death emerge and take shape (Neimeyer et al., 2014). Making sense of this experience can be a difficult challenge that involves not only the family but also professionals and the community (Gandino et al., 2019). For families who have experienced a SIDS death, the grieving process can be complex and related to multiple factors: for the mother, for example, the presence of intraindividual vulnerabilities (Goldstein et al., 2019b), the timing of the loss, and the ongoing attachment process (Rubin, 1984), while others are related to the developmental stage of the family life cycle and the family relationships in place at the time of the loss (Walsh and McGoldrick, 2004, 2013).

In the process of adjustment to loss, the family system should aim to fulfill two tasks: on the one hand, to provide the opportunity to come into contact with death and share the experience of loss; on the other hand, to help the family invest in new relationships or existential goals, with the goal of supporting the griever individually but leading the family to a new internal reorganization (Walsh and McGoldrick, 2013). In addition, it is important that the pain of these couples and families be endured both in the immediate aftermath of the loss and in the long term: Indeed, it has been observed that the effects of this pain can impair future attachment relationships and lead to the emergence of “paradoxical parenting” (Warland et al., 2011). So, it would be important that new studies also be conducted on the experiences of all family members who have suffered the loss.

## 6. Limitations, strengths, and clinical implications

This article aims to highlight the international literature on the psychological consequences of the sudden and unexpected death of a child, not only for the mother and the couple, but also for the other family members. It was decided to approach this topic with a broader focus of attention, in order to capture the impact that these tragic losses have outside the parent couple. In this sense, it was possible to observe that not only the parents suffer this loss, but also the other family figures around them, such as the grandparents and the siblings already present.

Compared to the considerable amount of data on mothers’ experiences, there is no corresponding number of studies on fathers. In fact, there is only one study focusing on male partners in the Māori population, which is also difficult to generalize due to the strong cultural and social component; other works include the father figure in studies of couples. We can say that further studies focusing exclusively on the paternal experience are needed to better legitimize the pain resulting from this loss and to provide the basis for support tailored to men’s specific needs. Moreover, surveys of siblings are not based on a direct examination of their behavior, but on the impressions of parents, particularly mothers. Based on the analysis of sibling surveys, it is important to conduct *ad hoc* studies that examine the real-life experiences of siblings at specific ages and to develop guidelines to assist parents in communicating about death and grief with siblings. In addition, grandparenting and comparative studies of stillbirth, neonatal and SIDS deaths are not new. Findings about grandparenting experiences and hypotheses about differences in grief between stillbirth, neonatal death, and SIDS are based on old studies; therefore, we believe there will be an opportunity to review and update these data in light of new studies.

No medical databases were considered in the review of the literature; instead, it was decided to examine only the psychological databases. Therefore, it is advisable to deepen the investigation and consider databases from other disciplines.

The studies included in this review point to the need for an intervention that targets the needs of each family member and is tailored to the specifics of SIDS loss, rather than more generalized grief. The role of the psychologist must become more important and central in the moments following the loss to make the best use of available resources and reduce risk factors as much as possible. Until medical science finds the causes of this syndrome, clinical psychology must strive to develop programs that address the experiences of these families with continuity and competence, offering them a pair of lenses better suited to see the eclipse that has obscured their existence and preparing them to reinvest in desire and life.

## Data availability statement

The original contributions presented in this study are included in the article/supplementary material, further inquiries can be directed to the corresponding author.

## Author contributions

GG had the idea of this work and contributed to the selection of included studies. AD, AS, and EV performed the bibliographic search, created the dataset, and contributed to writing of the manuscript. CC, SF, and FV resolved the methodological doubts of possible studies and helped in the first version of this manuscript. GDF collaborated in methodological aspects of the manuscript and revised the manuscript. All authors read the manuscript and agreed with its submission.
